# Supplementary material for: Navigating the microbial community in the trachea-oropharynx of breast cancer patients with or without neoadjuvant chemotherapy (NAC) via endotracheal tube: has NAC caused any change?
Source: PeerJ. 2023 Nov 23;11:e16366. doi: 10.7717/peerj.16366 (PMC10676715; doi:10.7717/peerj.16366)
Supplement: Supplemental Information 1 — Abbreviations: NAC, Neoadjuvant chemotherapy [file peerj-11-16366-s001.docx]

| **Sample Name** | **category** | **Chao1** | **Shannon** | **Inverse Simpson** |
| --- | --- | --- | --- | --- |
| N1 | NAC | 235.4 | 5.591929 | 0.947094 |
| N2 | NAC | 160.2 | 5.320615 | 0.95842 |
| N4 | NAC | 175.5 | 5.111982 | 0.934454 |
| N5 | NAC | 180.1 | 4.344428 | 0.885182 |
| N6 | NAC | 96.0 | 0.79962 | 0.14371 |
| N7 | NAC | 225.0 | 4.679606 | 0.926747 |
| N8 | NAC | 229.2 | 4.809828 | 0.923381 |
| N9 | NAC | 208.3 | 5.247425 | 0.926697 |
| N10 | NAC | 197.3 | 5.515966 | 0.952589 |
| N11 | NAC | 133.0 | 5.515826 | 0.967829 |
| N12 | NAC | 177.5 | 5.2058 | 0.942339 |
| N13 | NAC | 98.0 | 5.025063 | 0.945054 |
| N14 | NAC | 267.1 | 5.649062 | 0.953355 |
| N15 | NAC | 202.2 | 5.528603 | 0.963003 |
| N16 | NAC | 168.3 | 5.174627 | 0.937772 |
| N17 | NAC | 107.5 | 4.529947 | 0.926505 |
| N18 | NAC | 329.5 | 6.158155 | 0.962489 |
| N19 | NAC | 242.5 | 5.723479 | 0.960096 |
| N20 | NAC | 301.7 | 5.568461 | 0.936216 |
| N21 | NAC | 179.8 | 4.285863 | 0.870112 |
| C1 | Non-NAC | 307.9 | 6.387112 | 0.975044 |
| C2 | Non-NAC | 135.0 | 5.061953 | 0.937559 |
| C3 | Non-NAC | 242.5 | 5.670409 | 0.963185 |
| C4 | Non-NAC | 226.5 | 5.775189 | 0.961388 |
| C5 | Non-NAC | 213.2 | 5.668634 | 0.957199 |
| C6 | Non-NAC | 225.5 | 5.262244 | 0.941774 |
| C7 | Non-NAC | 255.3 | 5.732199 | 0.960906 |
| C8 | Non-NAC | 214.5 | 5.493915 | 0.947316 |
| C9 | Non-NAC | 235.2 | 5.822885 | 0.964121 |
| C10 | Non-NAC | 299.8 | 6.065216 | 0.971481 |
| C11 | Non-NAC | 242.0 | 5.642067 | 0.961811 |
| C12 | Non-NAC | 211.1 | 5.28082 | 0.949935 |
| C13 | Non-NAC | 224.5 | 5.228643 | 0.935318 |
| C14 | Non-NAC | 194.2 | 5.999308 | 0.972398 |
| C17 | Non-NAC | 256.5 | 6.09556 | 0.971097 |
| C18 | Non-NAC | 299.8 | 6.000124 | 0.96779 |
| C19 | Non-NAC | 152.1 | 4.827913 | 0.93859 |
| C20 | Non-NAC | 229.1 | 5.563657 | 0.952745 |
| C21 | Non-NAC | 261.1 | 5.762178 | 0.952672 |
| C22 | Non-NAC | 178.0 | 5.166178 | 0.926212 |

Abbreviations: NAC, Neoadjuvant chemotherapy
