# Supplementary material for: Navigating the microbial community in the trachea-oropharynx of breast cancer patients with or without neoadjuvant chemotherapy (NAC) via endotracheal tube: has NAC caused any change?
Source: PeerJ. 2023 Nov 23;11:e16366. doi: 10.7717/peerj.16366 (PMC10676715; doi:10.7717/peerj.16366)

**BLAST+ processing of multiple top hits**

After conducting a BLAST+ search for the top 5 results of each ASV sequence, the best hit with the highest Bit score was selected. However, if a given result did not satisfy both Query Coverage >= 85 and Identity Percentage >= 85, taxonomy assignment was not carried out. In cases where the Bit score was tied, the result with the lower E-value was chosen. If the E-values were the same, the result with the longer alignment length was adopted.

**DADA2 parameters**

DADA2 truncLen was set to Forward 250bp and Reverse 200bp

Sequences with an expected error greater than 2 were removed using maxEE=2.

Other parameters were set to default values.

**Negative control sample quality control result**

DNA concentration: 0.62 ng/µL

Total amount: 0.022 µg

Library QC: concentration: 0 ng/µL

TapeStation D1000 Screen Tape

**Comparison of diversity indices between clinical stages by NAC/non-NAC using Mann-Whitney test**

Within NAC group; Stage 2 (n = 9) and Stage 3 (n = 11)


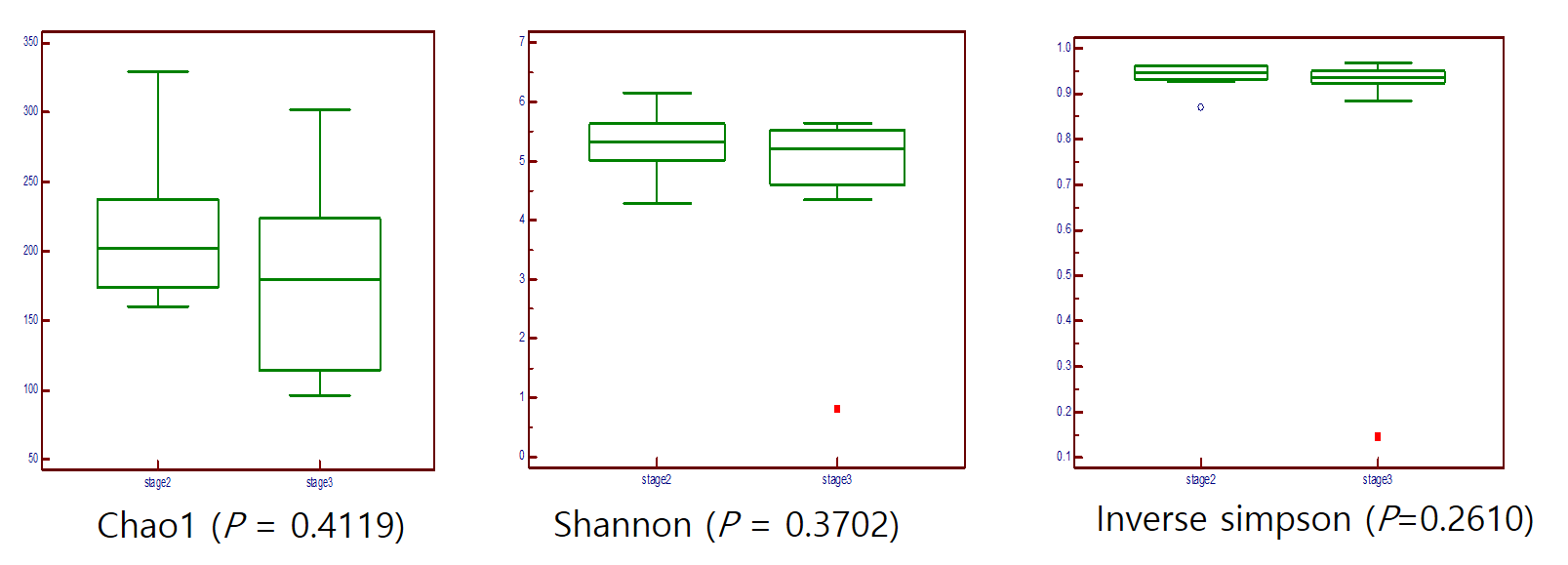


Within non-NAC group; Stage 1 (n = 12) and Stage 3 (n = 8)


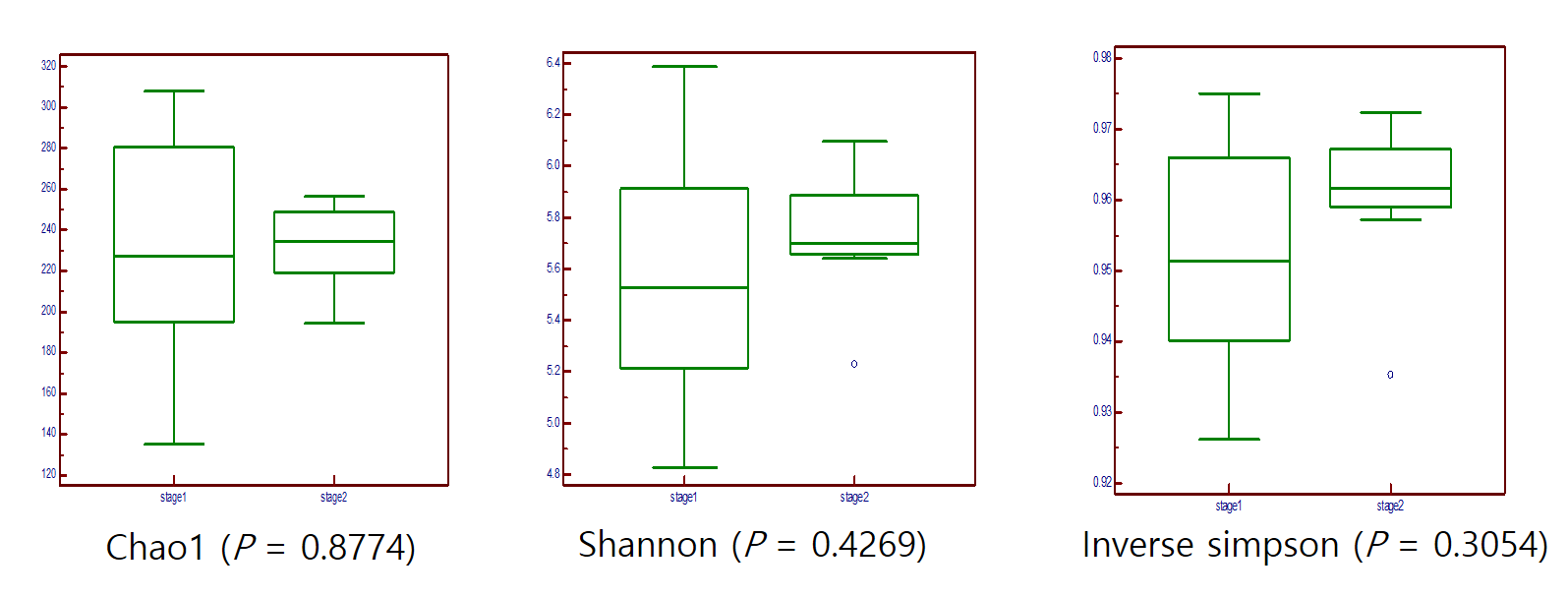

Supplement: Supplemental Information 2 [file peerj-11-16366-s002.docx]
