# Supplementary material for: Navigating the microbial community in the trachea-oropharynx of breast cancer patients with or without neoadjuvant chemotherapy (NAC) via endotracheal tube: has NAC caused any change?
Source: PeerJ. 2023 Nov 23;11:e16366. doi: 10.7717/peerj.16366 (PMC10676715; doi:10.7717/peerj.16366)
Supplement: Supplemental Information 4 [file peerj-11-16366-s004.pdf]

Within NAC group; stage2 vs stage 3

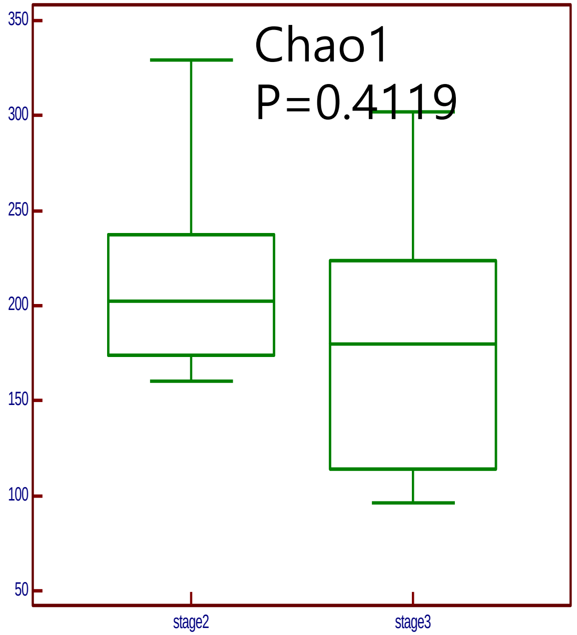

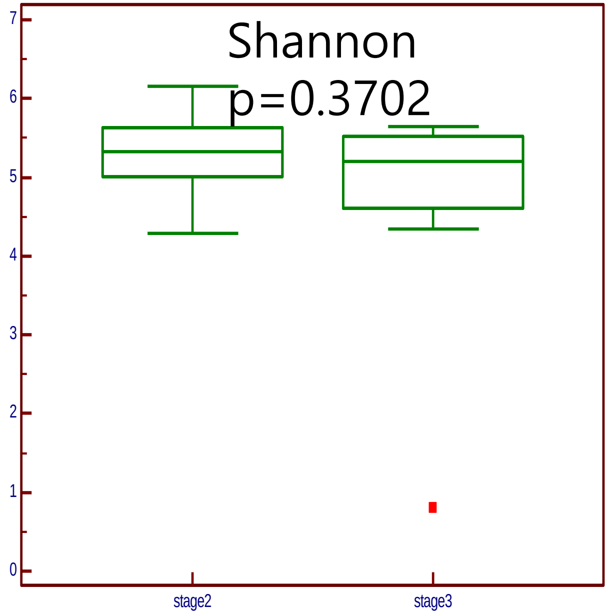

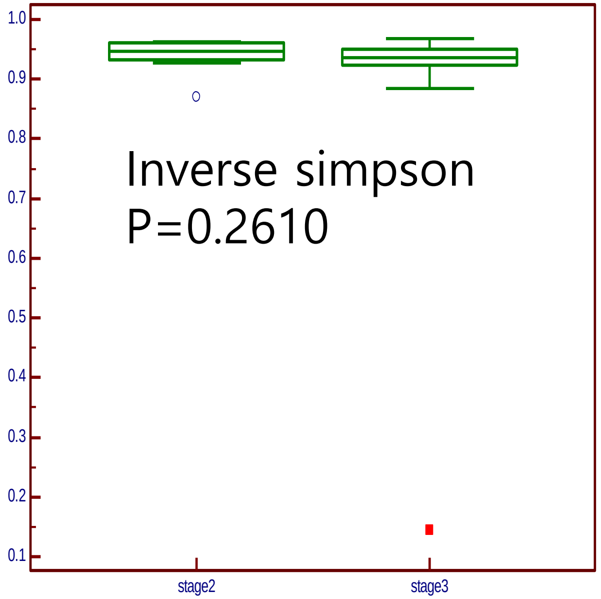

Mann-Whitney test (independent samples)

Sample 1	
Variable	stage2
Sample 2	
Variable	stage3

	Sample 1	Sample 2
Sample size	9	11
Lowest value	160.2000	96.0000
Highest value	329.5000	301.7000
Median	202.2000	180.1000
95% CI for the median	169.2934 to 241.5204	105.7929 to 236.0104
Interquartile range	173.7000 to 237.1750	113.8750 to 223.9750

Mann-Whitney test (independent samples)

Average rank of first group	11.7778
Average rank of second group	9.4545
Mann-Whitney U	38.00
Test statistic W	104.000
Two-tailed probability	P = 0.4119

Data comparison graph

Mann-Whitney test (independent samples)

Sample 1	
Variable	stage2
Sample 2	
Variable	stage3

	Sample 1	Sample 2
Sample size	9	11
Lowest value	4.2859	0.7996
Highest value	6.1582	5.6491
Median	5.3206	5.2058
95% CI for the median	4.7393 to 5.7053	4.4966 to 5.5254
Interquartile range	5.0039 to 5.6248	4.5999 to 5.5159

Mann-Whitney test (independent samples)

Average rank of first group	11.8889
Average rank of second group	9.3636
Mann-Whitney U	37.00
Test statistic W	103.000
Two-tailed probability	P = 0.3702

Mann-Whitney test (independent samples)

Sample 1	
Variable	stage2
Sample 2	
Variable	stage3

	Sample 1	Sample 2
Sample size	9	11
Lowest value	0.8701	0.1437
Highest value	0.9630	0.9678
Median	0.9471	0.9362
95% CI for the median	0.9278 to 0.9622	0.9165 to 0.9527
Interquartile range	0.9325 to 0.9607	0.9242 to 0.9507

Mann-Whitney test (independent samples)

Average rank of first group	12.2222
Average rank of second group	9.0909
Mann-Whitney U	34.00
Test statistic W	100.000
Two-tailed probability	P = 0.2610

Data comparison graph

Within non-NAC group; stage1 vs stage 2

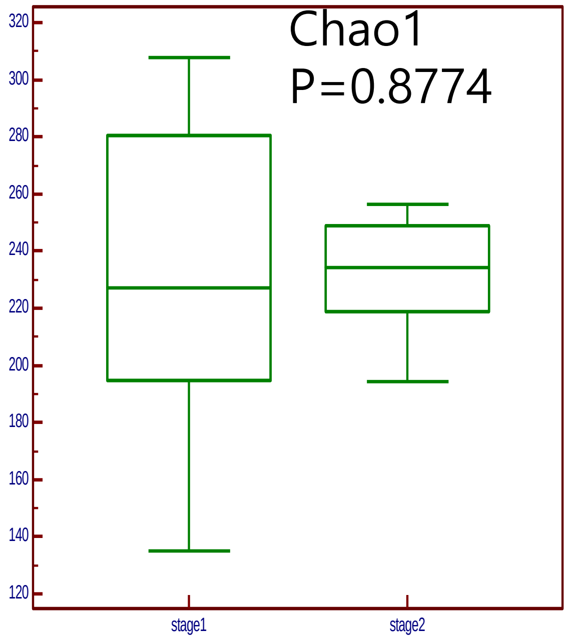

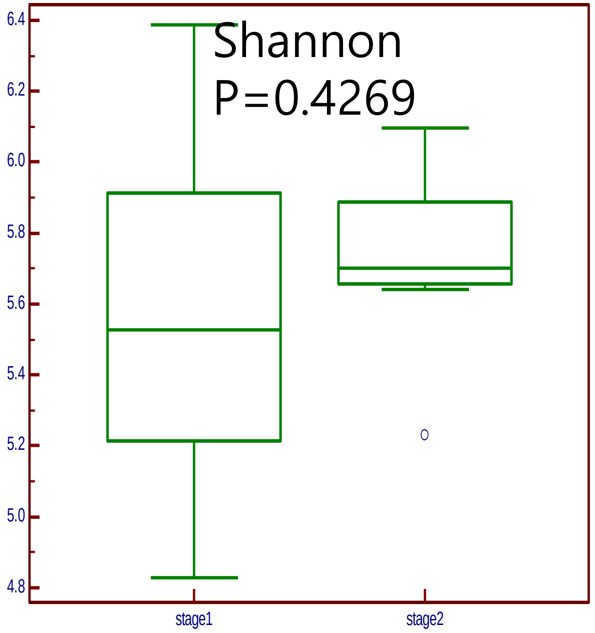

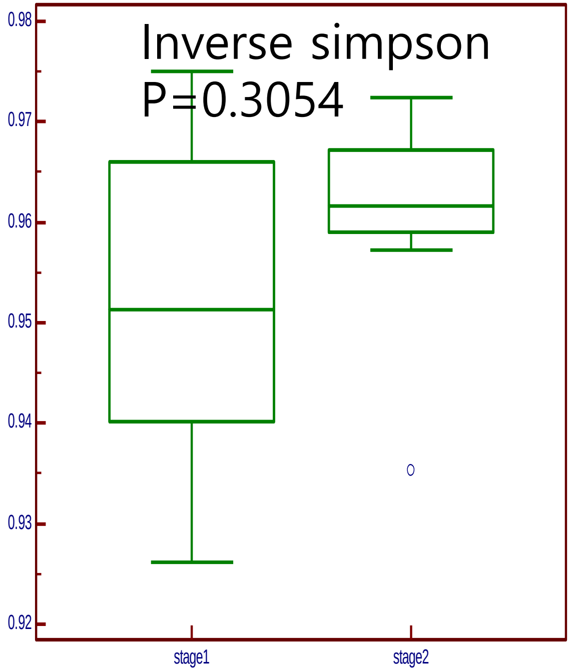

Mann-Whitney test (independent samples)

Sample 1	
Variable	stage1
Sample 2	
Variable	stage2

	Sample 1	Sample 2
Sample size	12	8
Lowest value	135.0000	194.2000
Highest value	307.9000	256.5000
Median	227.3000	234.2500
95% CI for the median	183.7190 to 293.1134	209.6040 to 255.5271
Interquartile range	194.5500 to 280.4500	218.8500 to 248.9000

Mann-Whitney test (independent samples)

Average rank of first group	10.3333
Average rank of second group	10.7500
Mann-Whitney U	46.00
Test statistic Z (corrected for ties)	0.154
Two-tailed probability	P = 0.8774

Data comparison graph

Mann-Whitney test (independent samples)

Sample 1	
Variable	stage1
Sample 2	
Variable	stage2

	Sample 1	Sample 2
Sample size	12	8
Lowest value	4.8279	5.2286
Highest value	6.3871	6.0956
Median	5.5288	5.7013
95% CI for the median	5.1828 to 5.9695	5.5638 to 6.0175
Interquartile range	5.2142 to 5.9115	5.6554 to 5.8872

Mann-Whitney test (independent samples)

Average rank of first group	9.5833
Average rank of second group	11.8750
Mann-Whitney U	37.00
Test statistic W	115.000
Two-tailed probability	P = 0.4269

Data comparison graph

Mann-Whitney test (independent samples)

Sample 1	
Variable	stage1
Sample 2	
Variable	stage2

	Sample 1	Sample 2
Sample size	12	8
Lowest value	0.9262	0.9353
Highest value	0.9750	0.9724
Median	0.9513	0.9616
95% CI for the median	0.9391 to 0.9672	0.9531 to 0.9713
Interquartile range	0.9402 to 0.9660	0.9591 to 0.9671

Mann-Whitney test (independent samples)

Average rank of first group	9.3333
Average rank of second group	12.2500
Mann-Whitney U	34.00
Test statistic W	112.000
Two-tailed probability	P = 0.3054

Data comparison graph
